# Acute Liver Failure in a Pediatric Patient with Tetralogy of Fallot following Cardiac Catheterization

**DOI:** 10.1155/2020/7537902

**Published:** 2020-07-26

**Authors:** Irim Salik, Jasmeet Easwar, Michael Jacoby

**Affiliations:** Department of Pediatric Anesthesiology at Westchester Medical Center, New York Medical College, Valhalla, NY, USA

## Abstract

A 4-month-old female with Tetralogy of Fallot (TOF) developed acute liver failure (ALF) following a cardiac catheterization procedure to correct severe stenosis of her right ventricular outflow tract (RVOT) conduit via balloon angioplasty. Cardiac history included TOF, heterotaxy syndrome, ipsilateral pulmonary veins, interrupted inferior vena cava (IVC) with azygos continuation, patent ductus arteriosus (PDA), and pulmonary atresia that was repaired with a right ventricle (RV) to pulmonary artery (PA) conduit. This is the first case described of its kind to our knowledge. Consent was obtained from the patient's family to publish this case report.

## 1. Introduction

ALF is defined as new onset coagulopathy, hepatic encephalopathy, and jaundice in a patient with no previous history of liver disease [1]. Patients classically present with an abnormal cholestatic biochemical profile including elevated alkaline phosphatase, total bilirubin, an increase in the international normalized ratio (INR), and occasionally, elevated transaminases.

Cardiac etiologies most commonly responsible for hepatic dysfunction include venous outflow obstruction leading to hepatic congestion or reduced cardiac output leading to impaired hepatic oxygen delivery. The hepatic vascular bed is supplied by a fixed cardiac output, and hemorrhagic centrilobular necrosis of the liver can result when hepatic oxygen delivery decreases to less than a critical threshold [2].

## 2. Description of the Case

This 4-month-old, 4 kg infant presented with repeated episodes of desaturation and increasing oxygen requirements in the setting of TOF, heterotaxy syndrome, ipsilateral pulmonary veins, interrupted IVC with azygous continuation, and pulmonary atresia. Surgical history included ventricle septal defect (VSD) patch placement, PDA ligation, and placement of a 9 mm RV to PA conduit 3 months prior to the patient's current presentation. During the original surgical repair, the right-sided pulmonary veins were not baffled to the left atrium, leading to a residual left to right cardiac shunt. The postoperative course was complicated by two failed attempts at extubation, ultimately requiring a tracheostomy and gastrostomy tube (g-tube) placement.

Currently, preintervention echocardiogram revealed systemic RV pressure at 75 mmHg, mild-moderate bilateral branch PA stenosis, mild right ventricular dilation, hypoplastic left atrium, and severe conduit stenosis (RVOT pressure gradient 93 mmHg). Right and left ventricular function were normal. Physical exam revealed a palpable liver 3 cm below the costal margin. It was surmised that the patient's recurrent desaturation was respiratory in nature, because a residual ASD or VSD that would enable right to left shunting was not found. The patient was referred for left and right cardiac catheterization to evaluate RVOT stenosis.

**Figure 1:**
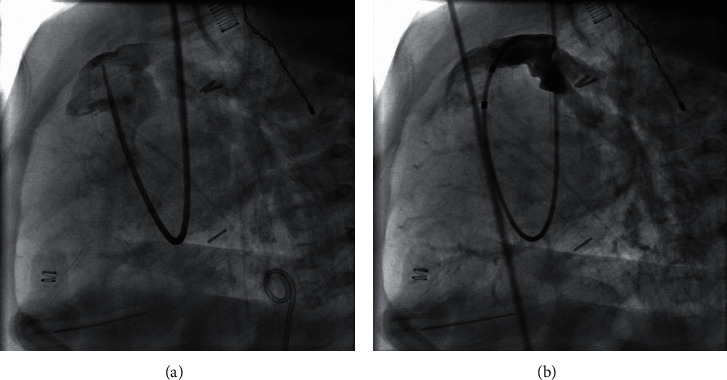
(a) RV to PA conduit stenosis preintervention. (b) RV to PA conduit s/p angioplasty.

Precatheterization vital signs included a blood pressure 70/40, pulse rate of 138, respiratory rate 22, and oxygen saturation 97% on a ventilator with FiO_2_ of 0.4. Lab results indicated a basic metabolic panel (BMP) within normal limits with glucose of 83. Preoperative EKG revealed normal sinus rhythm and a right bundle branch block with a QRS duration of 113 ms. On the morning of the procedure, g-tube feeds were replaced with pedialyte®. The intraoperative BMP revealed normal results with a blood glucose of 97. The central venous pressure (CVP) was recorded as 24 at the start of the procedure. The preintervention RV pressure was 75 mm Hg, RV-PA conduit gradient was 50 mm Hg, and the ratio of RV to LV pressure was 1.25 (see Table 1 for complete pre- and postcatheterization pressures). Balloon wedge catheter placement confirmed severe RVOT conduit stenosis. Balloon angioplasty of the RVOT conduit was performed with 10 atm of pressure intermittently for 4 minutes to reduce RV pressure and improve ventricular function. The case concluded within five hours with no significant intraoperative complications (see Figure 1 for angiographic images before and after balloon angioplasty).

Upon arrival to the pediatric intensive care unit (PICU), the patient was noted to have seizure-like activity. Finger-stick revealed a glucose concentration of less than 10 mg/dL, treated immediately with dextrose, leading to symptom resolution. Later that evening, the patient began bleeding from intravenous and central line insertion sites. Subsequent laboratory values revealed an aspartate aminotransferase level of 3753, alanine aminotransferase level of 2983, ammonia of 215, fibrinogen 109, INR 1.9, blood urea nitrogen 26, and creatinine 1.2. The patient was diagnosed with ALF, hepatic encephalopathy, and disseminated intravascular coagulation. An abdominal ultrasound revealed hepatomegaly with volume redistribution, concerning for portal hypertension secondary to right ventricular strain. The patient was treated with fresh frozen plasma, red blood cells, platelets, vitamin K, tranexamic acid, lactulose, and neomycin. Liver function improved with supportive care within 2 weeks. The patient subsequently developed an aneurysm of her PA conduit which required reoperation, but was discharged after a month-long stay in the PICU once synthetic hepatic function and hypertransaminasemia fully resolved.

## 3. Discussion

The liver is a highly vascular organ that receives up to 25% of an individual's total cardiac output. One quarter of this blood supply is fully oxygenated blood from the hepatic artery. The remainder of hepatic blood flow is from deoxygenated blood at venous pressure derived from the portal vein. Unfortunately, the portal vein is unable to autoregulate blood flow, making it dependent on mesenteric circulation, as well as the gradient between hepatic and portal venous pressures [3]. Following a 60% reduction in portal flow, the liver becomes susceptible to ischemic injury after the hepatic artery buffer response is fully taxed. The liver is a resilient organ; although sensitive to hemodynamic changes, it can successfully withstand short periods of ischemia. The most common cardiac induced acute liver injury is known as ischemic or hypoxic hepatitis, which likely occurred in this infant [4].

This critically ill infant likely presented to the cardiac catheterization laboratory with deficient glycogen stores and hepatic congestion due to chronically elevated right-sided cardiac pressures. The infant was unable to mount an appropriate physiological response to hypoglycemia given pre-existing hepatic compromise due to right heart strain as evidenced by an elevated CVP. Intraoperatively, ballooning the RVOT likely exacerbated hepatic congestion and compromised the cardiac output enough to cause a drastic increase in hepatic transaminases, as well as a short period of renal insufficiency. Impaired gluconeogenesis, along with a depleted catecholamine state and glycogen stores lead to the clinical presentation of seizure activity secondary to hypoglycemia.

The liver's most susceptible region to hypoxic or anoxic injury is known as Rappaport liver zone 3, in which reduced hepatic arterial perfusion can lead to centrilobular necrosis [5]. In the extreme case, right-sided heart failure can lead to reduced hepatic blood flow, elevated hepatic venous pressure, and decreased arterial saturation. In turn, sinusoidal edema and dilatation can cause hepatic atrophy and fibrosis [6]. In this patient, the combination of reduced arterial perfusion during ballooning of the RVOT and chronic passive venous congestion likely led to central hypoxia and subsequent ALF. Birrer et al. have reported that states of passive hepatic congestion may predispose hepatocytes to greater hypoxic injury secondary to hypotension [7]. After peaking at 1–3 days following hemodynamic injury, transaminase levels usually decrease by half within 72 hours and return to baseline within 7–10 days. This is reliant on the restoration of hepatic perfusion and resolution of cardiac insult in a timely fashion [8].

Although likely caused during intervention in the cardiac catheterization laboratory, the differential diagnosis for ischemic hepatitis and ALF includes systemic hypotension, drug-induced injury, and multiorgan dysfunction secondary to shock, sepsis, or cardiac disease. Patients with Budd–Chiari syndrome, veno-occlusive disease, and the abuse of vasoconstricting toxins such as cocaine and methamphetamine can also exhibit ALF [8]. There are a number of systems in place to score liver failure, including the Kings College Hospital Criteria, (KCHC), the Clichy score, the Model for End-Stage Liver Disease (MELD) score, and the Pediatric End-Stage Liver Disease (PELD) score, although none are well associated with patient outcome [9]. The Liver Injury Unit (LIU) score has been developed specifically for pediatric ALF and includes peak total bilirubin, prothrombin time (PT) or INR, and ammonia level [10].

In an infant with existing hepatic compromise undergoing a catheterization procedure that can further restrict cardiac output, it is important to be aware of and monitor for ALF. More frequent monitoring of blood glucose concentration and open communication with the cardiac interventionalist regarding risk stratification is imperative to avoid hepatic insult.

## Figures and Tables

**Table 1:** : Pre- and postcatheterization values.

	Precatheterization data	Postcatheterization data
Central venous pressure	21 mmHg	23 mmHg
Main pulmonary artery pressure (conduit pressure)	75 mmHg	45 mmHg
Right ventricular systolic pressure	75 mmHg	60 mmHg
Right ventricular end-diastolic pressure	13 mmHg	9 mmHg
Right ventricular-pulmonary artery conduit gradient	50 mmHg	25 mmHg
Right ventricular/Left ventricular pressure ratio	1.25	1
